# Serum levels of vitamin D and tumour necrosis factor-alpha in adults with metabolic syndrome

**DOI:** 10.15584/ejcem.2021.4.3

**Published:** 2021-12-30

**Authors:** Sheu Kadiri Rahamon, Olatunbosun Ganiyu Arinola, Mabel Ayebatonyo Charles-Davies, Kehinde Sola Akinlade, John Ayodele Olaniyi, Adesoji Adedipe Fasanmade, Oyediran Emmanuel Oyewole, Mayowa Ojo Owolabi, Jane Roli Adebusuyi, Olufunke Olayemi Hassan, Muhammed Babatunde Ajobo, Kehinde Adigun, Maria Onomaghuan Ebesunun, Omolara Olutosin Popoola, Wemimo Omiyale, Emmanuel Oluyemi Agbedana

**Affiliations:** 1 Department of Chemical Pathology, College of Medicine, University of Ibadan, Ibadan, Nigeria; 2 Department of Immunology, College of Medicine, University of Ibadan, Ibadan, Nigeria; 3 Department of Haematology, College of Medicine, University of Ibadan, Ibadan, Nigeria; 4 Department of Medicine, College of Medicine, University of Ibadan, Ibadan, Nigeria; 5 Department of Health Promotion and Education, College of Medicine, University of Ibadan, Ibadan, Nigeria; 6 Department of Medical Social Services, University College Hospital, Ibadan, Nigeria; 7 Dietetics Department, University College Hospital, Ibadan, Nigeria; 8 Department of Family Medicine, University College Hospital, Ibadan, Nigeria; 9 Department of Chemical Pathology, College of Health Sciences, Olabisi Onabanjo University, Ago-Iwoye, Nigeria

**Keywords:** inflammation, metabolic syndrome, vitamin D

## Abstract

**Introduction.:**

Reports continue to show that a significant association exists between serum vitamin D level and metabolic syndrome (MS)-associated inflammation. However, information on the serum levels of vitamin D and alterations in inflammation in different vitamin D status is presently lacking.

**Aim.:**

To determine the serum levels of vitamin D and TNF-α, and assess their possible relationship with gender in individuals with MS.

**Material and methods.:**

Sixty adults with MS and 40 controls were enrolled into this case-control study. Serum vitamin D and TNF-α levels were measured and participants stratified into different vitamin D status.

**Results.:**

None of the participants had vitamin D deficiency and the mean vitamin D level was similar in MS compared with the controls. However, TNF-α level was significantly higher in MS compared with the controls. Serum vitamin D level had significant inverse correlation with serum TNF-α level in MS. Also vitamin D level was significantly lower while TNF-α level was significantly higher in female-MS compared with the male-MS.

**Conclusion.:**

Adults with MS have elevated TNF-α level which appears to be associated with the serum level of vitamin D. Also, females with MS have low vitamin D level and this may exacerbate the MS-associated inflammation in them.

## Introduction

Metabolic syndrome (MS) is a constellation of disorders which increases the propensity for developing various cardiometabolic diseases including cardiovascular diseases (CVD), insulin resistance (IR), and diabetes mellitus (DM).^1^ It is a major public health problem affecting approximately 30% of adults and 25% of the worldwide population.^2^ Genetic predisposition, lack of physical activity, increased consumption of high calorie-low fibre fast food, overweight and obesity underlie the aetiology of MS.^1,3^

Despite the availability of reports on MS preventive and therapeutic strategies, the prevalence of MS continues to rise worldwide even, in the developing world where poverty is widespread.^4–10^ This has been attributed to technological advances favouring sedentary lifestyle and non-adherence to therapeutic lifestyle changes (TLCs).

There is an avalanche of reports indicating that inflammation plays a vital role in the pathophysiology of MS.^11–14^ The reports of Alberti et al., Zimmet et al. and Grundy et al. showed that elevated circulated inflammatory markers such as tumour necrosis factor-alpha (TNF-α) and reduced levels of anti-inflammatory molecules are components of multiplex risk factors associated with MS.^15–17^ The altered inflammatory responses in MS favours pro-inflammation and it is initiated by positive energy balance, manifesting as central adiposity, which causes adipocyte hypertrophy and a dysregulation of adipokine secretory patterns resulting in an imbalance between pro- and anti-inflammatory adipokines production, and infiltration of the adipose tissue by macrophages.^11,13^

Prominent among the inflammatory peptides produced by the adipocytes and the adipose tissue infiltrating macrophages is TNF-α which has been shown to impair insulin signalling in insulin sensitive tissues.^13,18^ This results in insulin resistance (IR), one of the hallmarks of MS, which stands out as the main end point underlying the clustering of CVD risk factors in MS.^19,20^ TNF-α promotes IR via serine phosphorylation of insulin receptor substrate 1 (IRS-1), as against the usual tyrosine phosphorylation. This phosphorylation in serine prevents IRS activation by the insulin receptor, blunts downstream signalling and facilitates the degradation of IRS protein.^21–26^

Understanding the interplay between molecules with anti-inflammatory properties and disordered inflammatory responses in MS is of the essence in developing preventive and therapeutic approaches to MS. One of the substances with profound anti-inflammatory properties is vitamin D.^27^ It is a pleiotropic hormone with critical roles in health maintenance.^28^ It contributes to the regulation of the proliferation, differentiation and function of various cells of the immune system hence; plays important roles in the regulation of both the innate and adaptive immune system.^29^ Martens et al. reported that several cells of the immune system including neutrophil, monocytes, macrophages, B-cells and T-cells express vitamin D receptor (VDR) which directly or indirectly modulates their activities towards achieving immunity.^30^ Reports have shown that an inverse relationship exists between vitamin D and MS and that an optimal serum vitamin D level lowers the risk.^31–33^

Although the mechanisms by which vitamin D reduces inflammation remains poorly understood, the reported lower serum level of vitamin D in individuals with MS indicates the possible role of vitamin D deficiency in the pathogenesis of MS-associated pro-inflammation.^27,34,35^ Presently, there is the dearth of information on the vitamin D status of Nigerians with MS. Similarly, information on the possible interplay between the serum levels of vitamin D and TNF-α is presently lacking.

## Aim

To determine the serum levels of vitamin D and TNF-α, and assess their possible relationship with gender in individuals with MS.

## Materials and methods

### Study design

The study was a case-control study.

### Study population

A total of 100 participants were enrolled into this study. They include 60 patients with metabolic syndrome (MS) diagnosed using the International Diabetes Federation (IDF) criteria 36 and 40 apparently healthy adults randomly selected from the cohorts of traders involved in a study titled “Risk Assessment of Type 2 Diabetes Mellitus and Dementia in Nigerians with Metabolic Syndrome”.^37^ All the study participants had no history of cardiovascular disease, diabetes mellitus and were not on any medication as earlier reported.^38^

### Ethical consideration

Ethical approval was obtained from the University of Ibadan/University College Hospital (UI/UCH) Joint Ethics Review Committee. Also, informed consent was obtained from the study participants.

### Sample collection

Venous blood (10 ml) was obtained from each study participant and serum samples obtained were stored at −20°C until analyzed.

### Laboratory analyses

The serum levels of vitamin D was determined using HPLC as described by Kand’ár and Záková while the serum TNF-α level was determined using ELISA (Boster Biological Technology, USA) following the Manufacturer’s instruction.^39^

### Vitamin D classification

The serum vitamin D level was classified as reported by Holick.^40^ Vitamin D levels ≤20, 21 – 29, 30 – 150 and >150 ng/ml were considered as deficiency, insufficiency, sufficiency and intoxication respectively.

### Statistical analysis

Continuous variables were subjected to Kolmogorov-Smirnov Test of Normality and differences in means or medians of the variables were compared using the independent Student’s t-test or Mann Whitney U depending on the Gaussian distribution of the variable. Association between dichotomous variables was determined using the Fisher’s Exact test. Correlation between vitamin D and TNF-α was determined using the Spearman correlation. Results are presented as mean ± standard deviation, number (percentages) and median (interquartile range) as appropriate. *P*-values less than 0.05 were considered as statistically significant.

## Results

The vitamin D status of the study participants is shown in Table 1. None of the study participants had VDD. The proportion of MS patients with vitamin D sufficiency was similar to that of controls. Only 1 patient had vitamin D intoxication among the MS patients (Table 1).

**Fig. 1.**
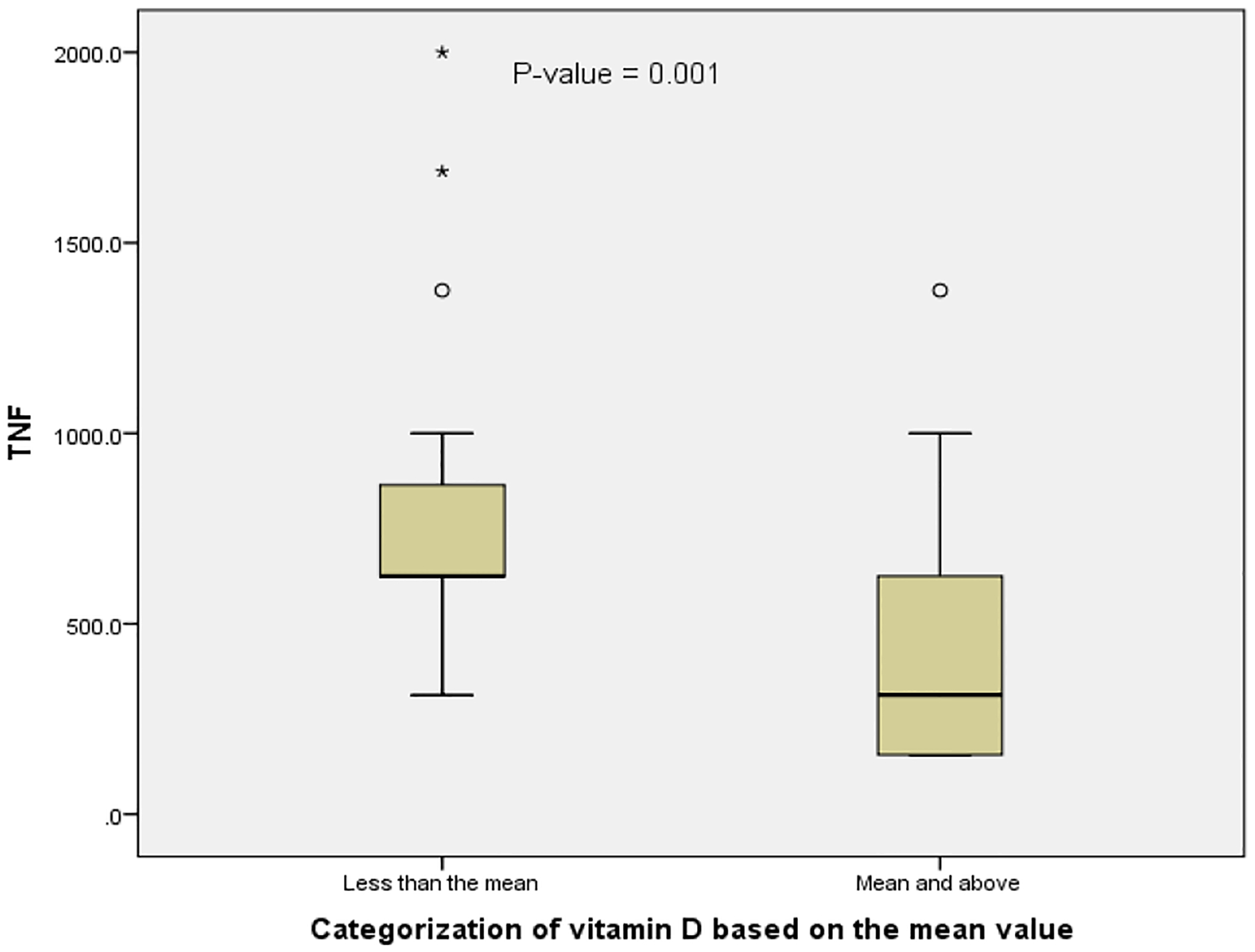
Serum TNF-α levels in MS patients with below or above the mean vitamin D level (37.70 ng/mL)

The possible effect of serum vitamin D concentration on serum TNF-α level was assessed by classifying the MS patients into two categories based on the mean level of vitamin D; 37.70 ng/mL. This classification became necessary as none of the patients had VDD and only a few had vitamin D insufficiency and intoxication. The median level of TNF-α was significantly higher in MS patients with vitamin D level lower than the mean compared with the MS patients with vitamin D level equal or greater than the mean (Fig. 1).

The median level of TNF-α was significantly higher in MS compared with the controls. However, the mean level of vitamin D was similar in both groups (Table 2).

**Fig. 2.**
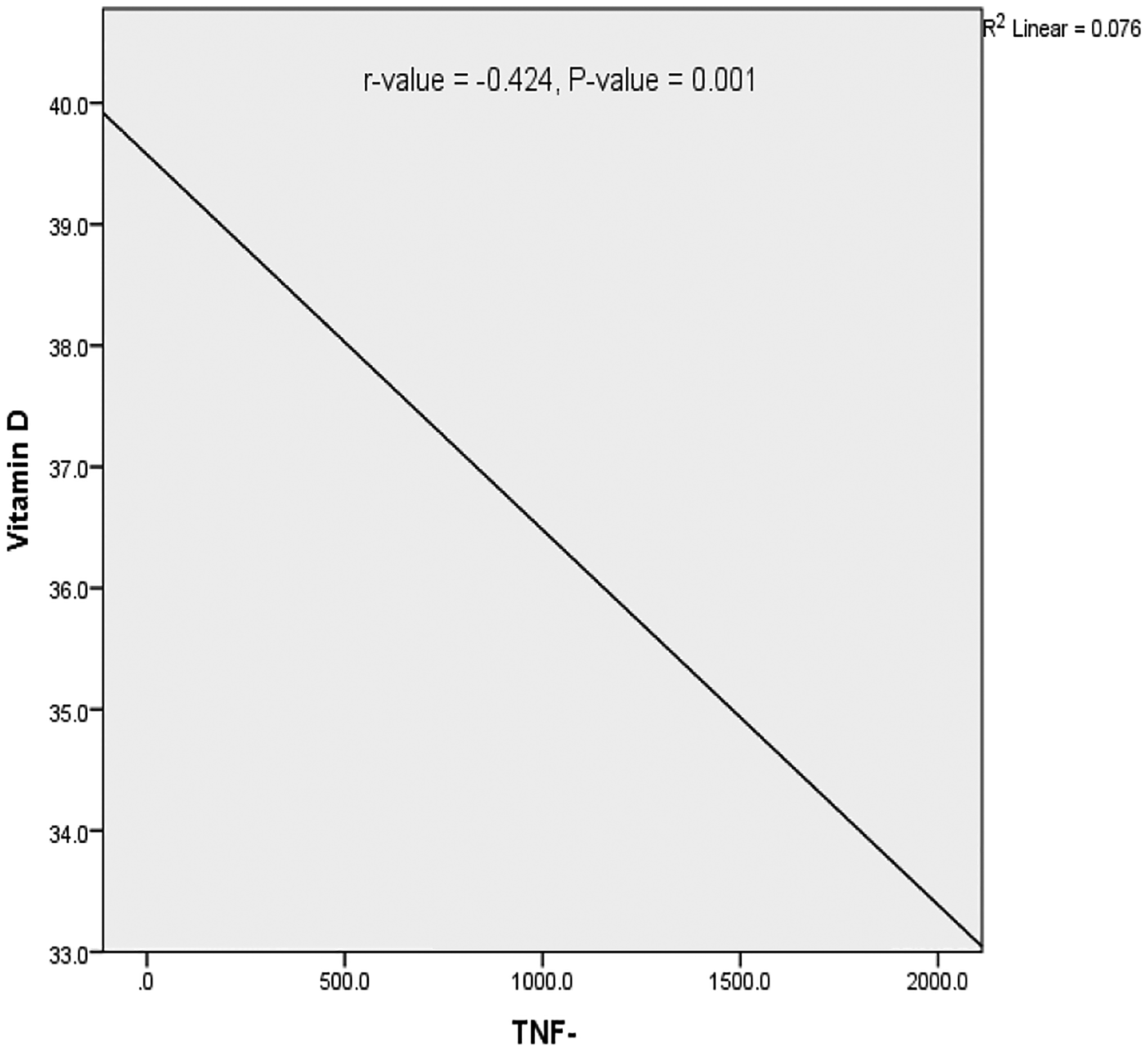
Correlation between serum levels of vitamin D and TNF-α

A significant inverse correlation was observed between vitamin D and TNF-α levels (Fig. 2).

Differences in the serum levels of vitamin D and TNF-α in MS based on gender is shown in Table 3. The mean serum level of vitamin D was significantly lower in females with MS compared with the males with MS. In contrast, the median serum level of TNF-α was significantly higher in females with MS compared with the males with MS (Table 3).

## Discussion

Understanding the dynamics of MS-associated pro-inflammation in different vitamin D status could facilitate potential therapeutic modulation of cytokine systems in MS.

In this study, the proportion of MS patients with vitamin D sufficiency and the mean serum vitamin D level were observed to be similar to that of controls and no single case of VDD was observed. This observation contradicts the reports of Farrell et al. and Godala et al. which showed lower level of vitamin D in individuals with MS.^34,35^ Incongruities in our report and that of previous reports could be due to selection of study participants. In our study, the study participants were all traders whose skins are usually exposed to solar ultraviolet B (UVB) radiation as majority of them trade in open market space for hours daily. It is well known that cutaneous synthesis of vitamin D via solar UV radiation is the major source of vitamin D with only a small proportion derived from dietary intake.^41^ Alagöl et al. showed that vitamin D deficiency (VDD) is more prevalent among individuals with little sun exposure.^42^ Similarly, a Manchester study which used whole-body irradiation for 6 weeks to simulate summer sunlight exposure showed that UV radiation equivalent to 30 min of sunlight 3 times per week achieved vitamin D level of >50 nmol/L in >90% of the study participants.^43^

TNF-α is an immunomodulatory inflammatory cytokine with pleiotropic effects. It is produced by numerous cells including macrophages/monocytes, natural killer cells (NKCs), lymphocytes and adipocytes during inflammation. It performs diverse range of intracellular signalling.^44–47^ The observed elevated serum level of TNF-α in MS compared with the controls was in line with previous reports.^48–50^ This observation could be due to the heightened phagocytosis of necrotic adipocytes in the adipose tissue. It could also be attributed to MS-associated IR which results in dyslipidaemia and metabolic endotoxaemia favouring pro-inflammation.

In a bid to understand the effect of serum vitamin D level on serum level of TNF-α, MS patients were classified into 2 groups using the mean vitamin D level of the group as most of the patients were vitamin D sufficient and comparison of serum TNF-α level based on vitamin D status was impossible. The observed elevation in serum TNF-α level in the below mean group supports and the significant inverse correlation observed between vitamin D and TNF-α levels in MS support the reports of Kayaniyil et al. and Milovanovik et al. who reported high level of TNF-α in their vitamin D deficient study participants.^51,52^ Our observations, which confirm the established anti-inflammatory properties of vitamin D, indicate that vitamin D could serve therapeutic purpose in MS and this could be mediated via upregulation of MAPK phosphatase-1 resulting in the inhibition of lipopolysaccharide (LPS)-induced p38 activation and cytokine production by monocytes/macrophages.^27^

Reports have shown that gender differences exist in vitamin D status with female gender negatively associated with vitamin D sufficiency.^53,54^ The observed higher vitamin D level in males with MS compared with the females with MS corroborates the reports of Borissova et al. and Yan et al. showing significantly lower level of vitamin D in females compared with males.^55,56^ Our observation could be as a result of differences in the amount of subcutaneous fat between males and females with MS. Vitamin D is fat soluble and could be stored in large amounts in the subcutaneous adipose tissue thereby, causing a reduction in the circulating level.^57^ Several reports have shown that women have more subcutaneous fat than men.^37,58,59^ Similarly, our observation could be due to differences in lifestyle and outdoor activities. Furthermore, the common use of sunscreen, which absorbs UVB, among women, could be partly responsible for our observation. Faurschou et al. showed that the use of sunscreen may lead to VDD as an exponential increase in serum vitamin D level was observed with decreasing thickness of sunscreen layer in response to UVB exposure.^60^

The observed low vitamin D level in females with MS could be responsible for the observed elevated level of TNF-α in females with MS compared with the males with MS. This observation further confirms the inverse relationship between vitamin D and TNF-α.

## Conclusion

It could be concluded from this study that adults with MS have elevated TNF-α level which appears to be associated with the serum level of vitamin D. Furthermore, females with MS have low vitamin D level and this may exacerbate the MS-associated inflammation in them. Therefore, females with MS may benefit from vitamin D supplementation.

## Figures and Tables

**Table 1.** Vitamin D status of the study participants

	MS	Controls	n	χ^2^	*P*-value
Vitamin D deficiency	0 (0.0%)	0 (0.0%)	0	2.835	0.242
Vitamin D insufficiency	2 (3.3%)	0 (0.0%)	2		
Vitamin D sufficiency	58 (96.7%)	39 (97.5%)	97		
Vitamin D intoxication	0 (0.0%)	1 (2.5%)	1		

**Table 2.** Serum levels of vitamin D and TNF-α in MS and controls

	MS (n = 60)	Controls (n = 40)	*P*-value
Vitamin D (ng/mL)	37.70 ± 4.32	37.96 ± 3.02	0.742
TNF-α (pg/mL)	625.00 (313.00 – 728.20)	312.50 (156.00 – 312.50)	<0.001*

* Significant at P<0.05,

MS = Metabolic syndrome, TNF-α = Tumour necrosis factor-alpha

**Table 3.** Serum levels of vitamin D and TNF-α in males and females with MS

	Males (n = 15)	Females (n = 45)	*P*-value
Vitamin D (ng/mL)	40.87 ± 1.92	36.64 ± 4.39	0.000*
TNF-α (pg/mL)	156.00 (156.00 – 312.50)	625.00 (625.00 – 1000.00)	0.000*

* Significant at P<0.05,

MS = Metabolic syndrome, TNF-α = Tumour necrosis factor-alpha

## Data Availability

Data available in the repository of the Department of Chemical Pathology, College of Medicine, University of Ibadan.
